# Development and usability testing of *Understanding Stroke*, a tailored life-sustaining treatment decision support tool for stroke surrogate decision makers

**DOI:** 10.1186/s12904-020-00617-x

**Published:** 2020-07-20

**Authors:** Emily P. Chen, Cynthia Arslanian-Engoren, William Newhouse, Diane Egleston, Savina Sahgal, Aneesha Yande, Angela Fagerlin, Darin B. Zahuranec

**Affiliations:** 1 Center for Bioethics and Social Sciences in Medicine, University of Michigan Medical School, Ann Arbor, USA; 2 Department of Health Behavior and Biological Sciences, University of Michigan School of Nursing, Ann Arbor, USA; 3 Center for Health Communications Research, University of Michigan Institute for Healthcare Policy and Innovation, Ann Arbor, USA; 4 University of Michigan, Ann Arbor, USA; 5 Department of Population Health Sciences, University of Utah School of Medicine, Salt Lake City, USA; 6 Salt Lake City VA Informatics Decision-Enhancement and Analytic Sciences (IDEAS) Center for Innovation, Salt Lake City, USA; 7 Department of Neurology, University of Michigan Medical School, Ann Arbor, USA

## Abstract

**Background:**

Surrogate decision makers of stroke patients are often unprepared to make critical decisions on life-sustaining treatments. We describe the development process and key features for the *Understanding Stroke* web-based decision support tool.

**Methods:**

We used multiple strategies to develop a patient-centered, tailored decision aid. We began by forming a Patient and Family Advisory Council to provide continuous input to our multidisciplinary team on the development of the tool. Additionally, focus groups consisting of nurses, therapists, social workers, physicians, stroke survivors, and family members reviewed key elements of the tool, including prognostic information, graphical displays, and values clarification exercise. To design the values clarification exercise, we asked focus groups to provide feedback on a list of important activities of daily living. An ordinal prognostic model was developed for ischemic stroke and intracerebral hemorrhage using data taken from the Virtual International Stroke Trials Archive Plus, and incorporated into the tool.

**Results:**

Focus group participants recommended making numeric prognostic information optional due to possible emotional distress. Pie charts were generally favored by participants for graphical presentation of prognostic information, though a horizontal stacked bar chart was also added due to its prevalence in stroke literature. Plain language descriptions of the modified Rankin Scale were created to accompany the prognostic information. A values clarification exercise was developed consisting of a list of 13 situations that may make an individual consider comfort measures only. The final version of the web based tool (which can be viewed on tablets) included the following sections: general introduction to stroke, outcomes (prognostic information and recovery), in-hospital and life-sustaining treatments, decision making and values clarification, post-hospital care, tips for talking to the health care team, and a summary report. Preliminary usability testing received generally favorable feedback.

**Conclusion:**

We developed *Understanding Stroke*, a tailored decision support tool for surrogate decision makers of stroke patients. The tool was well received and will be formally pilot tested in a group of stroke surrogate decision makers.

**Trial registration:**

ClinicalTrials.gov (NCT03427645).

## Background

Patients hospitalized with serious acute illness face critical decisions about their medical care and use of life sustaining treatments. In neurologic illness such as stroke, patient decisional capacity is commonly impaired and decisions regarding interventions such as resuscitation, ventilation, or artificial nutrition are made by a surrogate decision maker who is typically a family member or trusted loved one. However, many surrogates are unprepared to make these decisions for their loved ones [1–3]. They may have difficulty determining what the patient would want, and as a result, may suffer long-term adverse psychological outcomes [3–6].

Decision support tools have been shown to improve the quality of decision making and reduce decisional conflict in multiple clinical settings [7]. However, only a limited number of tools have been developed to support decision-making on life sustaining treatment during an acute hospitalization [6, 8–13], and none have been specifically developed for stroke decision making. To address these gaps, we developed a web-based decision support tool, called *Understanding Stroke: A Guide for Family Decision Makers*, designed to help surrogate decision makers set the overall goals of treatment for a loved one hospitalized with an acute stroke. Here, we describe the development process, key features, and initial usability testing for this tool.

## Methods

### Patient and family advisory council

A Patient and Family Advisory Council (PFAC) was formed to provide continuous input on the development of the tool. The Council included stroke survivors and family members of survivors recruited from the patient population of the study center. The Council met at the beginning of the study in December 2017 to share their experiences with stroke with the study team, and also give feedback on specific proposed elements of the decision support tool. These tool elements included graphical displays of prognostic information (pie charts, bar charts, and pictographs/icon arrays); simplified descriptions of a standardized stroke outcome scale (modified Rankin scale) [14–17]; and a list of valued life activities to include in a values clarification exercise [18]. Subsequently, members of the PFAC provided ongoing feedback to the study team on draft versions of the materials and participated in up to two usability testing sessions during the final stages of development.

### Stakeholder focus groups

Focus groups of key stakeholders were recruited between December 2017 and April 2018. Groups were segmented intentionally to encourage candid responses. The four groups were as follows: nurses, therapists, and social workers with expertise in stroke or palliative care; physicians with expertise in stroke; stroke survivors and family members; and family members without stroke patients present. Provider participants were selected through a convenience sample from two hospital systems. Stroke survivors and their families, as well as bereaved family members, were recruited by contacting subjects from past research studies who agreed to be contacted for future research, as well as by sending out invitations to the Michigan Medicine Patient and Family Centered Care (PFCC) Program. Each focus group lasted approximately 2 h, and was primarily facilitated by author CA, a cardiovascular nurse researcher with extensive experience in conducting focus group sessions, and assisted by authors DZ, the principal investigator and a stroke physician, and WN, an expert in human computer interaction. Providers were asked about their experiences communicating stroke prognoses with patients and families, whether they use numbers or percentages and why. Stroke survivors and family members were asked about their experiences talking with the doctors and nurses early in the hospitalization about prognosis. Specifically, they were asked who discussed this with them, how the information was communicated and whether it was helpful, and what they think patients and families should have been told early on in the hospitalization. All groups provided feedback on draft elements on the tool, including graphical displays, descriptions of prognostic information, the language used to describe stroke outcome categories from the modified Rankin scale, and the list of valued life activities for the values clarification exercise. Focus group conversations were audio recorded and key themes were summarized by the study team. Demographic data for the focus group participants are listed in Additional file 1.

### Content development

**Fig. 1:**
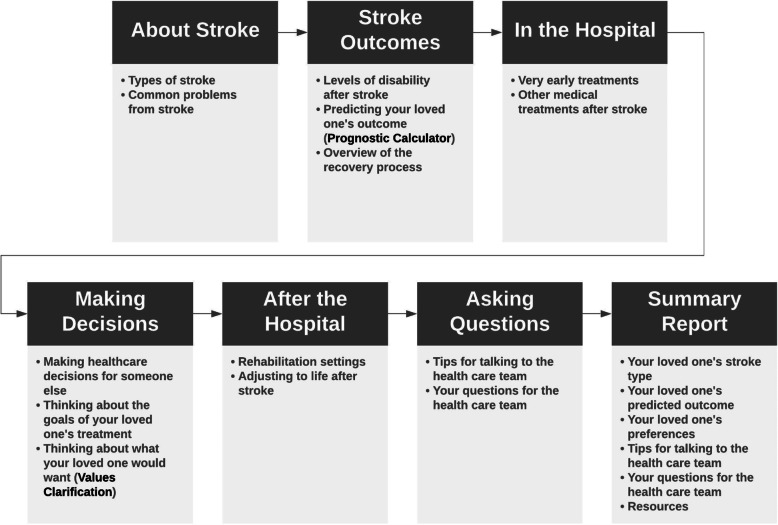
Content map. This shows the order in which each topic is presented in the *Understanding Stroke* tool. The user is encouraged to follow this sequence, but may choose to skip to any section using the navigation bar on the top of the page

After the focus groups were complete, the study team began developing the tool content. A content map was developed by a multi-disciplinary team including experts in vascular neurology, cardiovascular nursing, psychology, human computer interaction, and tailored behavioral change and health education interventions. The tool content was divided into 6 main sections – About Stroke (general introduction), Stroke Outcomes (levels of disability and prognostic calculator), In the Hospital (common treatments), Making Decisions (goals of treatment and values clarification), After the Hospital (rehabilitation options), and Asking Questions. Developing the content map was an iterative process where the organization of topics was rearranged to achieve the best flow, with the final content map shown in Fig. 1. Introductory descriptions of ischemic stroke and intracerebral hemorrhage were adapted from existing materials [19, 20]. A behavioral scientist with expertise in developing tailored health communication interventions revised the content to fit the goals of the decision support tool and ensured that it was written in plain language. Illustrations demonstrating ischemic stroke and intracerebral hemorrhage were also developed. The unique elements of the tool (e.g. prognostic calculator and values clarification), were written in more detail than the informational components, such as what rehabilitation options are available after leaving the hospital. Key information was then summarized into a printer-friendly summary report (see Additional file 2) that the surrogate decision maker could use to facilitate their communication with the treating health care team.

### Prognostic models and calculator

Ordinal prognostic models for *Understanding Stroke* were developed separately for ischemic stroke (IS) and intracerebral hemorrhage (ICH). We chose to develop a novel model rather than use an existing model as most existing models predict a binary outcome rather than a full ordinal range of outcomes after stroke [21, 22]. Detailed statistical methods for model development will be reported separately [23]. Briefly, data for the model development was taken from the Virtual International Stroke Trials Archive Plus (VISTA-Plus) [24] which has 90-day mRS data for 9904 IS patients and 890 ICH patients. The mRS was selected as the outcome based on its broad availability in stroke studies and the large number of cases with data available in VISTA-Plus. Common predictor variables for both IS and ICH include age, NIH Stroke Scale (NIHSS), history of atrial fibrillation, diabetes, and prior stroke. For ischemic cases, other predictors include the NIHSS Level of Consciousness score (item 1a from the NIHSS) and whether the patient received intravenous tissue plasminogen activator (IV tPA), while the ICH model included sex as a predictor variable. When a surrogate is enrolled, the study team manually entered predictor variables into the website’s study team dashboard, which then calculated the results and fed the information to the *Understanding Stroke* website. As the direct display of prognostic calculator information to surrogates was a novel aspect of this tool, it was designed with an option to disable the display of calculator information at the discretion of the investigators. This option was created to guard against the possibility of concerns about model accuracy for individual patients with unusual features at presentation, or in the event of negative feedback from early enrollees.

### Values clarification exercise

Medical decision making experts generally recommend the inclusion of values clarification methods in decision support tools to help elicit values relevant to the decision [18, 25]. We conceptualized the values clarification exercise in this setting as helping the surrogate to think about which abilities are essential for the patient’s quality of life.

To begin developing the values clarification exercise, a preliminary list of important activities of daily living and functional abilities was created by adapting items from other tools [26, 27] and by adding individual items suggested by the focus group participants. Duplicative or similar items were merged, and the study team added a few items common in post-stroke patients (e.g., “Could not move one side of their body”) to make a comprehensive list. To assess the relative importance of each item and help to assure readability, the comprehensive list was made into a card sorting exercise. A convenience sample of volunteer participants was recruited from outpatient stroke and cardiovascular clinic waiting areas. Participants were shown, in random order, a list of the 14 items that may be important for people to think about when making medical decisions for a family member. The participant was then asked to rank the items on a board in order from most important to least important and add any additional items they felt were missing from the pool. Any item that did not play a role in decision making was asked to be left off the board. Therefore, the number of items ranked varied for each participant. We recorded their responses and scored the items according to their rankings.

### Usability testing

Finally, two rounds of usability testing were completed with volunteers from the PFAC, focus group participants, and health care providers. In these sessions, participants were first asked to locate specific pieces of information or complete certain tasks within the tool. Then they were asked a few questions from a modified Systems Usability Scale (SUS) [28] regarding the level of difficulty and complexity of using the tool. Lastly, they were asked a series of open-ended questions about what they liked the most, what could be improved, and whether they felt anything was missing from the website. Participants in the first round of usability testing also participated in the card sorting exercise mentioned above.

## Results

A total of four focus groups were held that included 8 physicians, 1 nurse, 5 occupational or physical therapists, 1 social worker, 5 stroke survivors, and 6 family members. When asked about the presentation of prognostic calculator results, some providers, patients, and family members expressed that some people will like numbers, while others may find it distressing and unhelpful. It was suggested that a preface would be needed to warn surrogates about the potential emotional distress it may evoke and also to offer the option to not view the personalized numeric prognosis. Some stroke family members expressed the desire for the prognostic model to show outcomes beyond 3 months. However, due to limitations of the available source data, we were unable to accommodate this request. To clarify to the user that continued improvement is possible beyond 3 months, the following sentence was added – “While people can continue to improve for a year or more after a stroke, doctors have found that most recovery happens in the first 3 to 6 months.”

When asked about the best graphical format for presentation, pie charts were overwhelmingly favored by patients, surrogates, and providers alike. Other options with positive responses included the vertical bar chart, icon array [29], and the horizontal stacked bar chart (primarily preferred by providers due to its common presence in stroke literature). Although icon arrays are a commonly recommended format to present numeric information [30, 31], most participants did not favor icon arrays, in part due the complexity of displaying multiple outcome categories. The study team ultimately decided to display both a pie chart and a horizontal bar chart, with the option to toggle between the two options depending on surrogate preference.

A plain language description of each level of the modified Rankin Scale was created to accompany the prognostic information to help with interpretation of the prognostic model. Due to the study targeting a moderate to severe stroke population, and to minimize the number of displayed categories, mRS scores of 0–2 were combined to become a single category of “mild disability or better”. The other scores were maintained as distinct categories – “moderate disability”, “moderately severe disability”, and “severe disability”, for mRS 3, 4, and 5, respectively. Examples of residual symptoms were listed to help family members understand the range of possible outcomes. The descriptions also included examples on the types of assistance they may need, e.g., not able to live alone, need help with daily activities, etc. The full description of text used for the modified Rankin scale is shown in Table 1.

**Table 1:** Plain language description of the modified Rankin Scale

Modified Rankin Scale score^a^	Scale	Description
0–2	Mild Disability or Better	• May have some mild symptoms of stroke, such as - Weakness - Numbness - Changes in thinking or speaking • Able to live on their own and manage daily activities (bathing, shopping, preparing or getting meals and managing finances)
3	Moderate Disability	• Not able to do all of the activities they could do before the stroke • May have difficulty thinking or speaking • May need help with some daily activities (bathing, shopping, preparing or getting meals, managing finances) • Able to walk without help from another person, but may need a cane or a walker
4	Moderately Severe Disability	• Need help with some daily activities (eating, bathing, dressing, toileting) • May have more severe difficulty thinking or speaking • Not able to walk without help from another person • May need a wheelchair • Not able to live alone
5	Severe Disability	• Need help with most or all activities (eating, bathing, walking) • Not able to sit up in bed without help • Not able to move from a bed to a chair without help • Do not have full control of bladder or bowel function • Not able to live alone • Need constant nursing care and usually live in a long-term nursing facility

^a^The mRS column is not shown in *Understanding Stroke*

Given the multitude of life-sustaining treatment decisions that a stroke surrogate decision maker may face (e.g. intubation, resuscitation, feeding tube), *Understanding Stroke* was designed to frame the decision as setting the overall goals of treatment rather than on any individual treatment or procedure. The overall goals of treatment were adapted from prior work and includes Life-Prolonging Treatment, Basic Treatment, and Comfort Measures Only [32]. Information about the three possible goals of treatment are summarized for surrogates as shown in Table 2. This section on making treatment decisions also includes education on what it means to be a surrogate decision maker and the importance of focusing on what their loved one would want [33].

**Table 2:** Summary of goals of treatment

	Life-Prolonging Treatment Goal: Keep alive as long as possible	Basic Treatment Goal: Maintain physical and mental functions	Comfort Measures Only Goal: Maximize comfort and relieve pain
Cardiopulmonary resuscitation (CPR) and defibrillation (electric shock to the heart)	X		
Breathing machine (ventilator)	X		
Intensive Care Unit (ICU) care	X		
Intravenous (IV) therapy	X	X	Sometimes used for pain
Hospitalization	X	X	Sometimes
Physical, occupational, or speech therapy	X	X	
Pain relief	X	X	X

A total of 24 participants were recruited (22 patients or family members, and 2 non-physician members of the stroke team) for the card sorting exercise to assist in developing the values clarification component. One item regarding the ability to drive was ranked as the least important by participants and was eliminated. No new items were added by the participants. The final list consists of 13 items shown in Table 3, which was made into a checklist for the surrogate to check off. The stem of the exercise was “My loved one might consider stopping treatment to extend life (and choose comfort measures only) if, for a few months or more, they” An additional option of “None of the above” was added for surrogates who think their family member would want to extend life despite all of these circumstances.

**Table 3:** Values clarification exercise

Select the most important items (up to 5) “My loved one might consider stopping treatment to extend life (and choose comfort measures only) if, for a few months or more, they …”	
Were not able to talk, but could still engage in non-verbal communication	☐
Had difficulty thinking of words or understanding others	☐
Were not able to breathe without the help of a machine	☐
Were not able to participate in important hobbies, social, or religious activities	☐
Had difficulty thinking clearly or making decisions (e.g. needed help managing finances)	☐
Needed help from another person to eat, bathe, or take care of basic bathroom needs	☐
Were not able to live on their own or take care of themselves	☐
Needed a walker or wheelchair to move around	☐
Had to stay in bed constantly	☐
Needed a feeding tube to get nutrition	☐
Had a lot of discomfort or pain	☐
Had to stay in a nursing home or rehabilitation facility	☐
Could not move one side of their body	☐

Two rounds of usability testing were conducted. The first was done 6 months prior to making the tool available to participants to allow time to make changes, and the second was 1 month prior for a final check. The combined participants across both rounds included 2 stroke survivors, 5 family members, and 2 stroke providers. In the task directive section, most people were able to find a specific section when asked. For anything that was more difficult to find, interface and navigation enhancements were made to the tool to improve the visibility of these items to the user. Results from the modified SUS show that most people felt the website was easy to navigate and interact with. A small number of participants felt they had to learn a lot of things before they could start using the website in the first round, or that using the tool was at a moderate level of difficulty rather than easy. Participants liked the layout, the information presented, the presentation of the prognostic information, and the values clarification exercise. Some overall suggestions included making navigation easier by repositioning certain buttons and adding a website content outline and additional resource links to the main page. An overview of the final version of the tool content and graphics is shown in Additional file 2.

## Discussion

We described the development of *Understanding Stroke: A Guide for Family Decision Makers*, a web-based decision support tool for surrogate decision makers of stroke patients. The tool was developed through a rigorous process that involved a PFAC, four focus groups, a multidisciplinary study team, card sorting by volunteer patients and family members, and two rounds of usability testing. The tool received positive feedback during usability testing, and most individuals found the content helpful and the website easy to use. The text was rated as an 8th grade reading level in the Flesch-Kincaid readability test.

The tool features tailored content that takes into account the patient’s stroke type and medical history, and displays information and prognostic calculator results accordingly. To be mindful of family members’ preferences for information, the tool also offers the option to not see the prognosis. Goals of treatment are explained in detail, and a values clarification exercise is available to help surrogate decision makers determine what the patient would want in accordance with their values. Lastly, the tool summarizes key information from the interactive components and compiles a report which can be printed out by the family member.

Previous decision support tools addressing decisions on life-sustaining treatments have primarily focused on advanced care planning [12, 27, 34, 35] with relatively few tools designed for use during an acute inpatient hospitalization. Other tools designed for use during an acute hospitalization have been developed for traumatic brain injury [36] and prolonged mechanical ventilation [9, 13, 37]. A recent pilot testing of a decision aid for prolonged mechanical ventilation suggested some improved outcomes, including lower physician-surrogate discordance for expected patient survival, greater comprehension, and improved quality of communication post-intervention [9]. However, a larger randomized trial of the web-based version did not confirm the benefits other than observing a small reduction in decision conflict in the intervention group [37]. Our tool shares many features with this web-based tool, including the utilization of a prognostic model and visual display of results, a values clarification exercise that focuses on goals of treatment decisions, additional questions for the care team, and a summary report that compiles key information. However, the differences in disease context, intended timeframe of use, and our different design of the values clarification exercise suggests a separate study of *Understanding Stroke* is still warranted. In a systematic review done by Witteman et al., it was noted that there is a wide variety of design features in current values clarification tools, and more work is needed to evaluate their effects [38, 39].

*Understanding Stroke* is currently being pilot tested in a non-randomized trial of 50 surrogate decision makers, with 25 each in a historical control group and intervention group. Enrollment of the control group began in February, 2018, concurrent with the development of *Understanding Stroke*. Participants completed a baseline survey and a 1-month follow-up survey. For those in the intervention group, the website is shown on a tablet computer immediately following the baseline survey, followed by a post-intervention survey with tool-specific questions. Registration of this trial can be found on ClinicalTrials.gov (NCT03427645) [40].

There are several limitations to the current version of *Understanding Stroke* worth noting. One is that the tool focuses primarily on informational needs and does not address the emotional needs of surrogate decision makers. Other studies have shown that surrogates of seriously ill individuals have considerable need for emotional support [37, 41–43]. However, this tool is in the early development stage. A future version could incorporate more comprehensive interventions that include both information and emotional support to family members. Secondly, the modified Rankin Scale was selected as the outcome because of its broad availability and use in stroke patients. There were concerns among the participants that the mRS would not convey the different types of disability, such as cognitive or speech impairment, and that different readers could interpret the information differently. Future work should address stroke prognostic models with a broader range of outcomes that may be more meaningful to patients and families. The tool also lacks a physician-facing component or direct integration into clinicians’ workflow. This design was by choice, in part to simplify the development and implementation of the initial version of the tool. Here again, a future version could be adapted to include a physician facing component or more direct integration into the clinical workflow. Lastly, although the tool was developed by following user-centered design principles, potential future enhancement could be realized through an experience-based co-design process that more actively involves participants in re-imagining the current decision aid with the support of a trained facilitator [44]. Such a redesign should take into consideration the overall outcomes of the current tool and seek to improve that experience.

## Conclusion

In summary, we developed *Understanding Stroke*, a tailored decision support tool for surrogate decision makers of stroke patients. The tool was well received during usability testing and will be pilot tested for preliminary assessment of acceptability, as well as its effect on surrogates’ understanding of prognosis and decisional self-efficacy in the clinical setting.

## Supplementary information

**Additional file 1.** Focus Group Demographic Data. Demographic data and characteristics of focus group participants

**Additional file 2 **Overview of *Understanding Stroke*. Screenshots of the *Understanding Stroke* tool content, graphics, and summary report

## Data Availability

Not applicable.

## Abbreviations

ICH: Intracerebral hemorrhage
IS: Ischemic stroke
IV tPA: Intravenous tissue plasminogen activator
NIHSS: National Institutes of Health Stroke Scale
PFAC: Patient and Family Advisory Council
PFCC: Patient and Family Centered Care
SUS: Systems Usability Scale
VISTA-Plus: Virtual International Stroke Trials Archive Plus
