# Supplementary material for: Development and usability testing of Understanding Stroke, a tailored life-sustaining treatment decision support tool for stroke surrogate decision makers
Source: BMC Palliat Care. 2020 Jul 20;19:110. doi: 10.1186/s12904-020-00617-x (PMC7370629; doi:10.1186/s12904-020-00617-x)
Supplement: Supplementary file 1 — Additional file 1. Focus Group Demographic Data. Demographic data and characteristics of focus group participants [file 12904_2020_617_MOESM1_ESM.pdf]

Appendix 1

Focus Group Demographic Data

Table 1: Providers (n=15)

**Characteristic**	**Median (Range) or Count (%)**
Age	39 (27-53)
Gender	
Male	5 (33.3)
Female	10 (66.7)
Race	
American Indian or Alaska Native	0 (0)
Asian or Asian American	1 (6.7)
Black or African American	0 (0)
Native Hawaiian or Other Pacific Islander	0 (0)
White or European American	12 (80)
Other	0 (0)
Unknown	2 (13.3)
Hispanic or Latino	
Yes	0 (0)
No	15 (100)
Self-rated Health	
Excellent	6 (40)
Very Good	9 (60)
Good	0 (0)
Fair	0 (0)
Poor	0 (0)
Years of experience caring for stroke patients	
0-5	3 (20)
6-10	6 (40)
11-15	3 (20)
>15	3 (20)
Primary role in working with stroke patients	
Therapist	5 (33.3)
Nurse	1 (6.7)
Social Worker	1 (6.7)
Physician	8 (53.3)

Table 2: Stroke survivors and Family Members (n=11)

Characteristic	Median (Range) or Count (%)
Age	62 (49-69)
Gender	
Male	4 (36.4)
Female	7 (63.6)
Race	
American Indian or Alaska Native	0 (0)
Asian or Asian American	0 (0)
Black or African American	1 (9.1)
Native Hawaiian or Other Pacific Islander	0 (0)
White or European American	10 (90.9)
Other	0 (0)
Unknown	0 (0)
Hispanic or Latino	
Yes	0 (0)
No	10 (90.0)
Unknown	1 (9.1)
Self-rated Health	
Excellent	3 (27.3)
Very Good	2 (18.2)
Good	4 (36.4)
Fair	1 (9.1)
Poor	1 (9.1)
Health Literacy (single-item assessment: "How confident are you in filling out medical forms by yourself?")	
Not at all (1)	0 (0)
A little bit (2)	0 (0)
Somewhat (3)	1 (9.1)
Quite a bit (4)	4 (36.4)
Extremely (5)	6 (54.5)
Stroke survivor	5 (45.5)
Family member of a stroke survivor or bereaved family member	6 (54.5)
