# Supplementary material for: Development and usability testing of Understanding Stroke, a tailored life-sustaining treatment decision support tool for stroke surrogate decision makers
Source: BMC Palliat Care. 2020 Jul 20;19:110. doi: 10.1186/s12904-020-00617-x (PMC7370629; doi:10.1186/s12904-020-00617-x)
Supplement: Supplementary file 2 — Additional file 2 Overview of Understanding Stroke. Screenshots of the Understanding Stroke tool content, graphics, and summary report [file 12904_2020_617_MOESM2_ESM.pdf]

Appendix 2

I. Welcome Page

The home page welcomes the surrogate decision maker to the website and gives an overview of the sections.

A “start here” tag is added to the first section to encourage the surrogate to view the website in order.

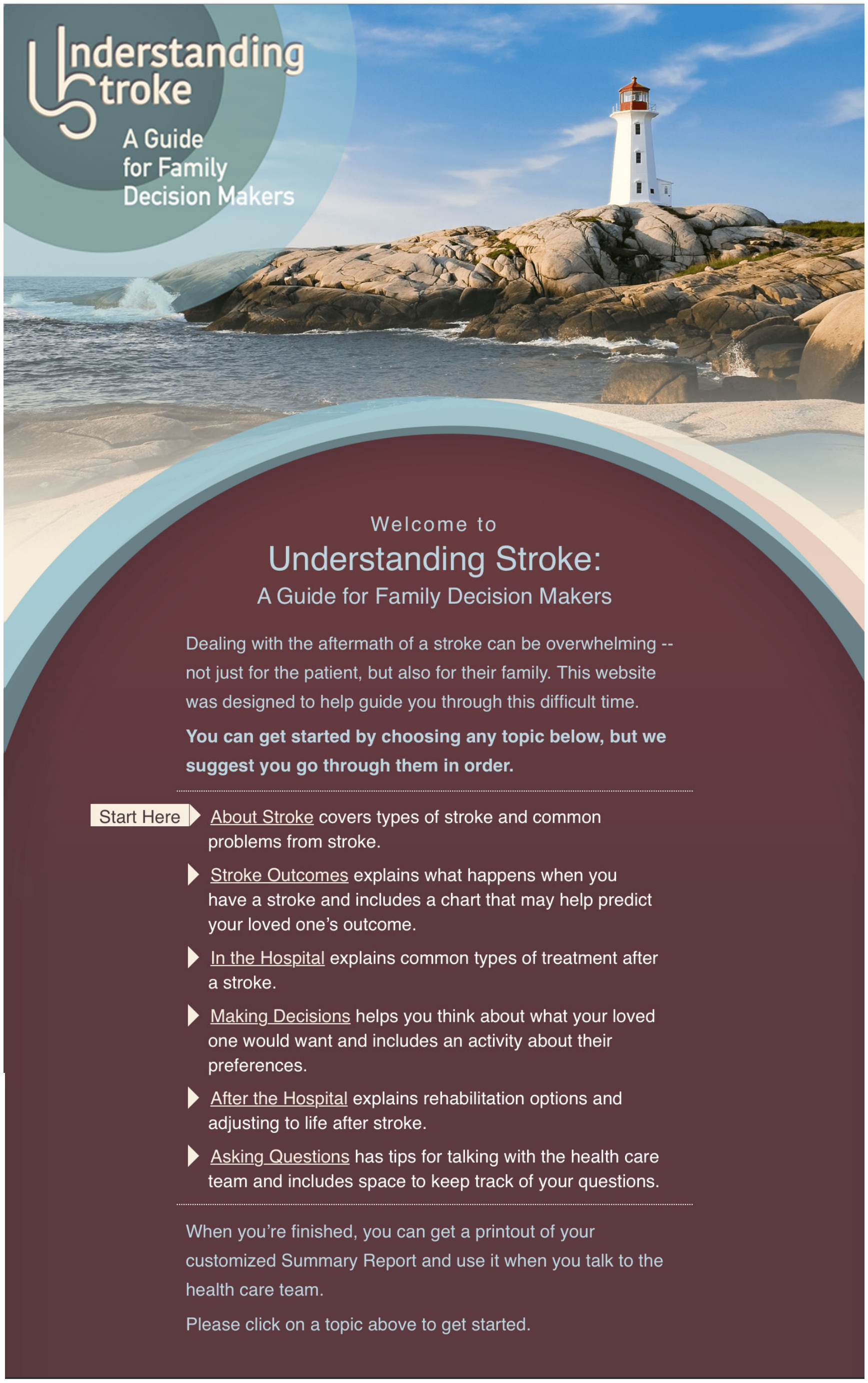

II. About Stroke

a. Types of stroke

The website starts by introducing the type of stroke the patient experienced. Depending on what type of the stroke the patient experienced, the surrogate will see one of the two versions of content shown to the right.

Example for Ischemic Stroke (IS)

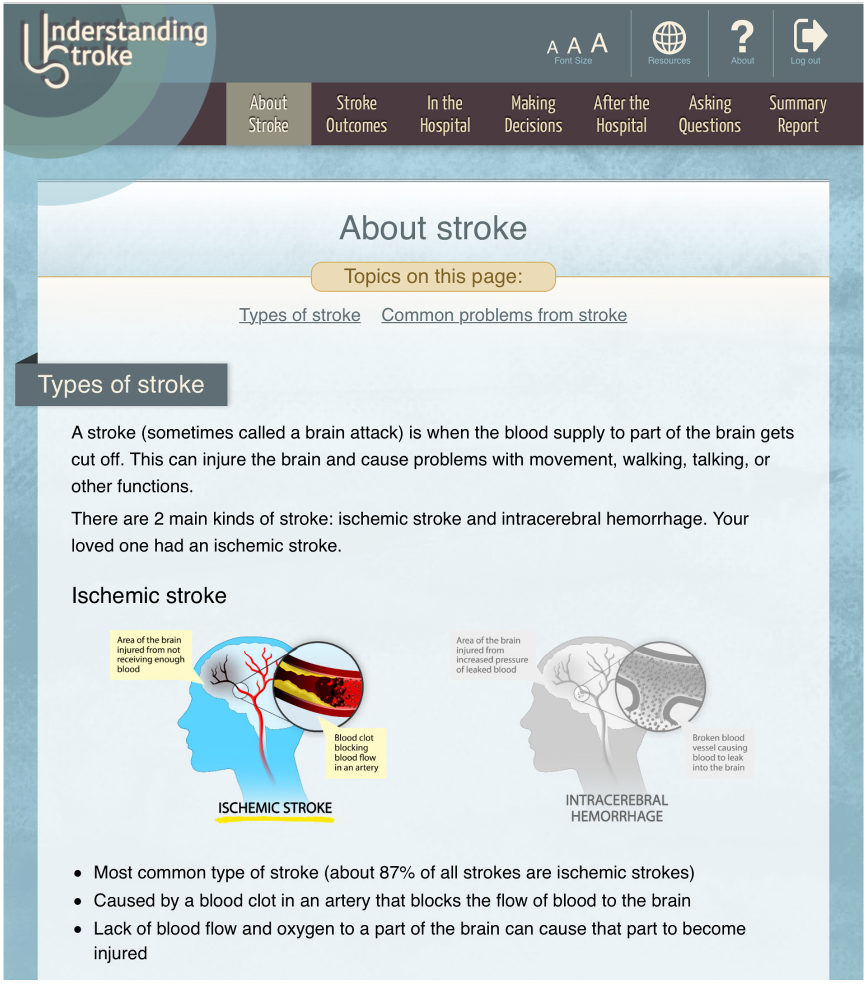

Example for Intracerebral Hemorrhage (ICH)

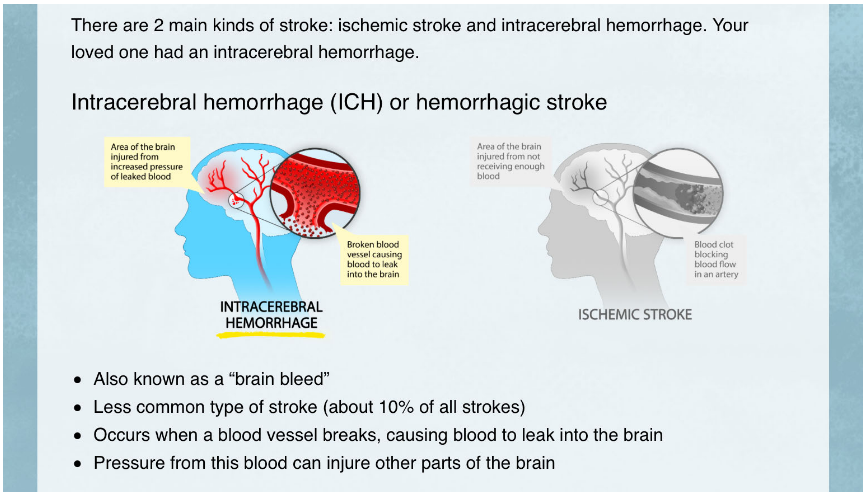

b. Common problems from stroke

Common symptoms that patients may experience after a stroke is listed here as well. This content is the same for IS and ICH.

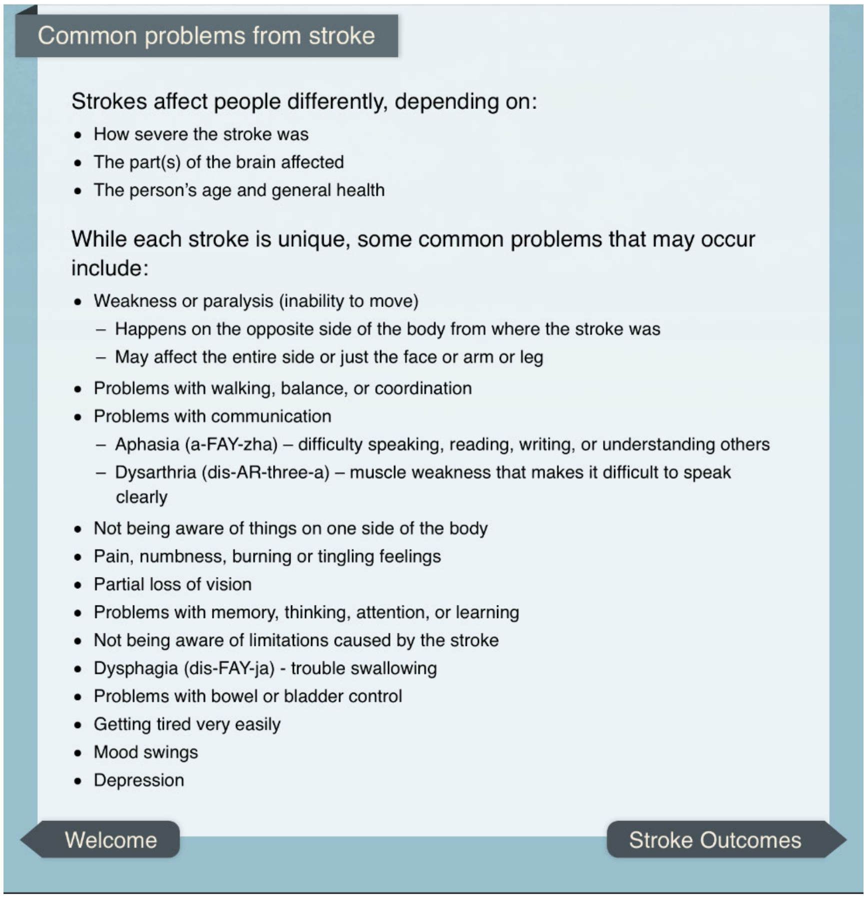

III. Stroke Outcomes

a. Levels of disability after stroke

A plain language description of each level of the modified Rankin Scale was created to accompany the prognostic information to help with interpretation of the prognostic model. Due to the study targeting a moderate to severe stroke population, and to minimize the number of displayed categories, mRS scores of 0-2 were combined to become a single category of "mild disability or better". The other scores were maintained as distinct categories – "moderate disability", "moderately severe disability", and "severe disability", for mRS 3, 4, and 5, respectively. Examples of residual symptoms were listed to help family members understand the range of possible outcomes. The descriptions also included examples on the types of assistance they may need, e.g., not able to live alone, need help with daily activities, etc.

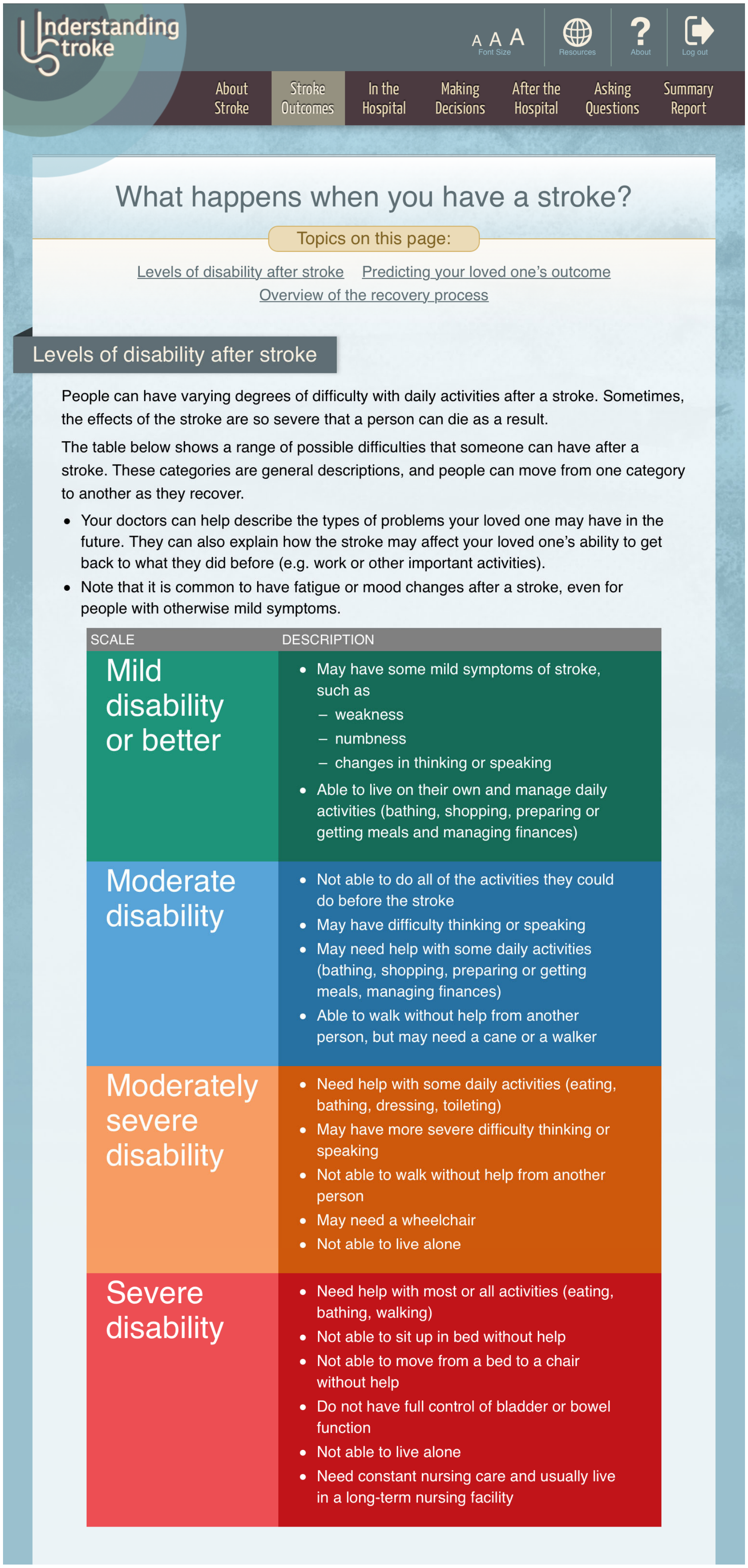

Understanding Stroke

A A A Font Size | Resources | About | Log out

About Stroke | Stroke Outcomes | In the Hospital | Making Decisions | After the Hospital | Asking Questions | Summary Report

What happens when you have a stroke?

Topics on this page:

Levels of disability after stroke | Predicting your loved one's outcome | Overview of the recovery process

Levels of disability after stroke

People can have varying degrees of difficulty with daily activities after a stroke. Sometimes, the effects of the stroke are so severe that a person can die as a result.

The table below shows a range of possible difficulties that someone can have after a stroke. These categories are general descriptions, and people can move from one category to another as they recover.

- Your doctors can help describe the types of problems your loved one may have in the future. They can also explain how the stroke may affect your loved one's ability to get back to what they did before (e.g. work or other important activities).
- Note that it is common to have fatigue or mood changes after a stroke, even for people with otherwise mild symptoms.

SCALE	DESCRIPTION
Mild disability or better	• May have some mild symptoms of stroke, such as<br>– weakness<br>– numbness<br>– changes in thinking or speaking<br>• Able to live on their own and manage daily activities (bathing, shopping, preparing or getting meals and managing finances)
Moderate disability	• Not able to do all of the activities they could do before the stroke<br>• May have difficulty thinking or speaking<br>• May need help with some daily activities (bathing, shopping, preparing or getting meals, managing finances)<br>• Able to walk without help from another person, but may need a cane or a walker
Moderately severe disability	• Need help with some daily activities (eating, bathing, dressing, toileting)<br>• May have more severe difficulty thinking or speaking<br>• Not able to walk without help from another person<br>• May need a wheelchair<br>• Not able to live alone
Severe disability	• Need help with most or all activities (eating, bathing, walking)<br>• Not able to sit up in bed without help<br>• Not able to move from a bed to a chair without help<br>• Do not have full control of bladder or bowel function<br>• Not able to live alone<br>• Need constant nursing care and usually live in a long-term nursing facility

b. Predicting your loved one's outcome

A brief introduction is given on the prognostic calculator, including what it is and how it works. Some surrogate decision makers in our focus groups expressed the desire for the model to show outcomes beyond 3 months. However, due to limitations of the source data, we were unable to accommodate this request. To clarify to the audience that continued improvement is possible beyond 3 months, a sentence was added to explain this. (See first red arrow)

Participants in our focus groups also suggested that some people may find numeric prognoses distressing rather than helpful. Therefore, a preface was added to warn surrogates about the potential emotional distress and give the option to not view the personalized numeric prognosis. (see second red arrow)

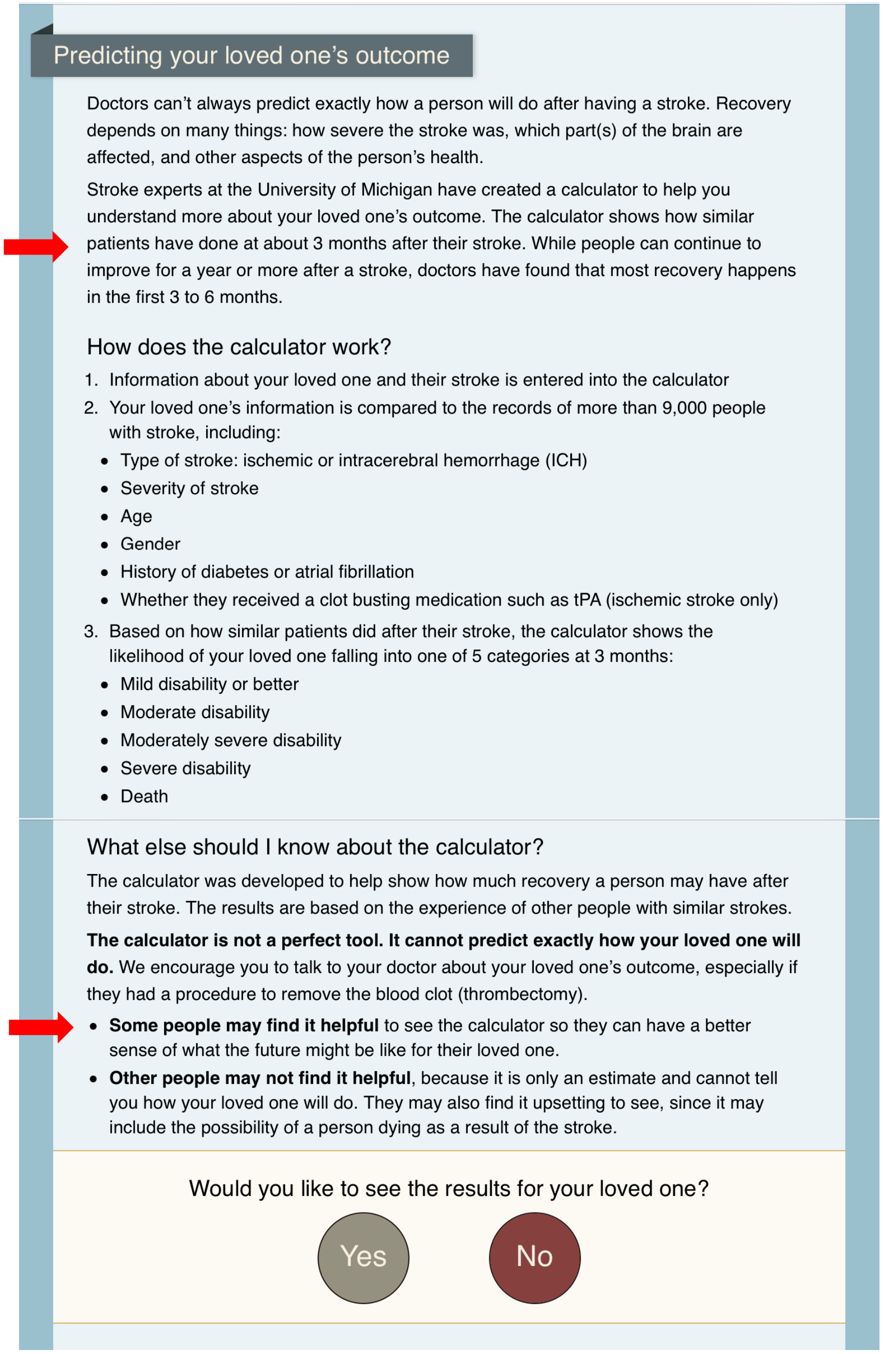
Predicting your loved one's outcome

Doctors can't always predict exactly how a person will do after having a stroke. Recovery depends on many things: how severe the stroke was, which part(s) of the brain are affected, and other aspects of the person's health.

Stroke experts at the University of Michigan have created a calculator to help you understand more about your loved one's outcome. The calculator shows how similar patients have done at about 3 months after their stroke. While people can continue to improve for a year or more after a stroke, doctors have found that most recovery happens in the first 3 to 6 months.

How does the calculator work?

1. Information about your loved one and their stroke is entered into the calculator
2. Your loved one's information is compared to the records of more than 9,000 people with stroke, including:
   - Type of stroke: ischemic or intracerebral hemorrhage (ICH)
   - Severity of stroke
   - Age
   - Gender
   - History of diabetes or atrial fibrillation
   - Whether they received a clot busting medication such as tPA (ischemic stroke only)
3. Based on how similar patients did after their stroke, the calculator shows the likelihood of your loved one falling into one of 5 categories at 3 months:
   - Mild disability or better
   - Moderate disability
   - Moderately severe disability
   - Severe disability
   - Death

What else should I know about the calculator?

The calculator was developed to help show how much recovery a person may have after their stroke. The results are based on the experience of other people with similar strokes.

The calculator is not a perfect tool. It cannot predict exactly how your loved one will do. We encourage you to talk to your doctor about your loved one's outcome, especially if they had a procedure to remove the blood clot (thrombectomy).

- **Some people may find it helpful** to see the calculator so they can have a better sense of what the future might be like for their loved one.
- **Other people may not find it helpful**, because it is only an estimate and cannot tell you how your loved one will do. They may also find it upsetting to see, since it may include the possibility of a person dying as a result of the stroke.

Would you like to see the results for your loved one?

Yes No

c. Graphical presentation of prognosis

We included the option to toggle between a pie chart, which was overwhelmingly favored by patients, surrogates, and providers alike, and a horizontal stacked bar chart, which is commonly shown when reporting stroke clinical trial results.

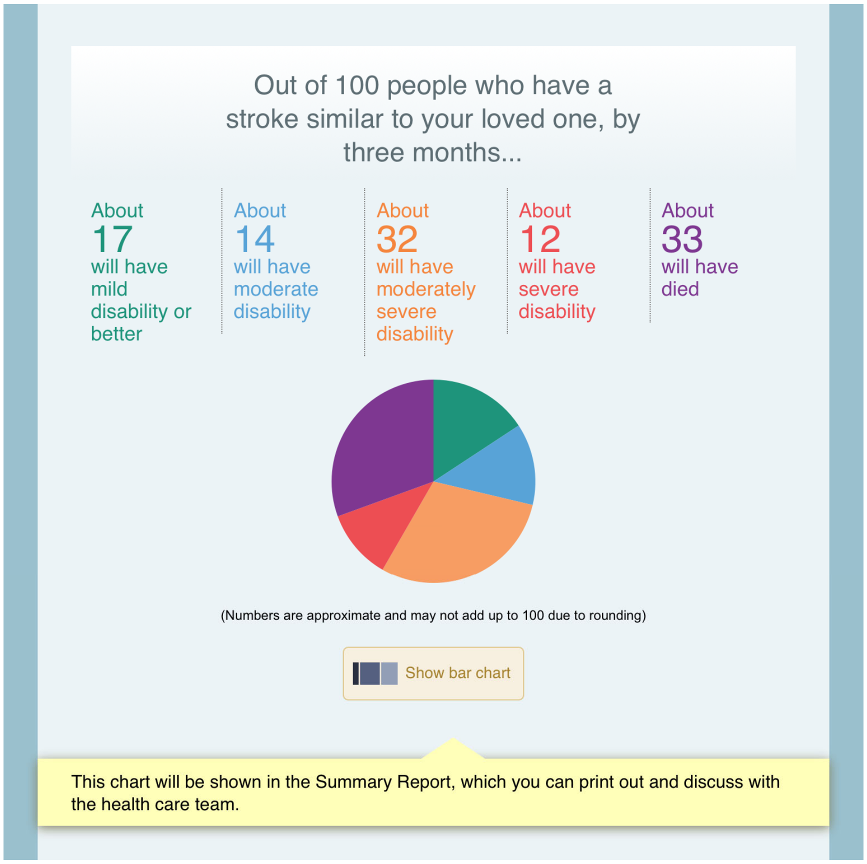

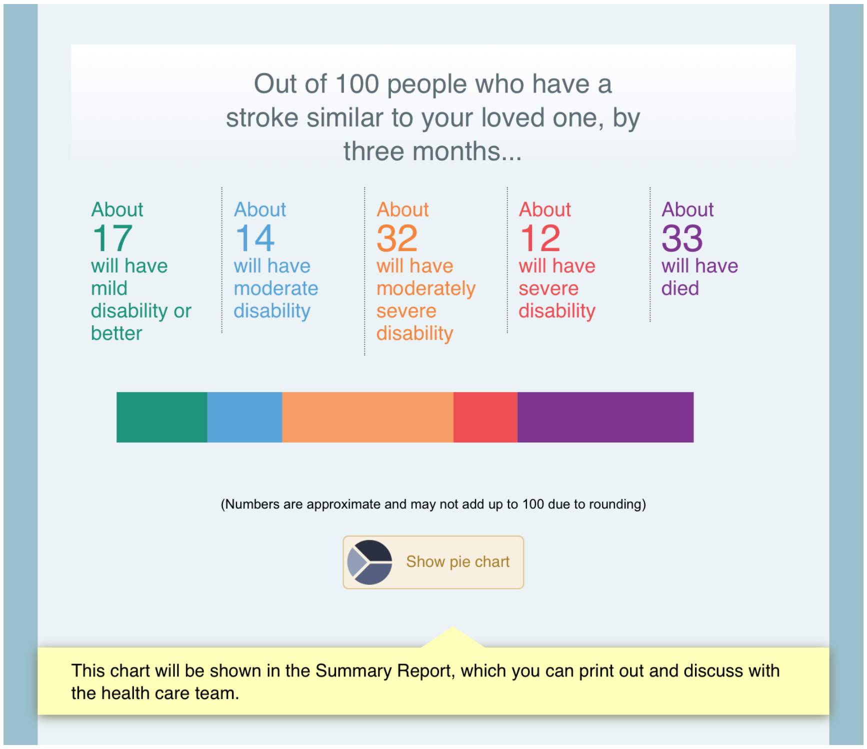

d. Overview of the recovery process

A brief overview is given on the recovery process.

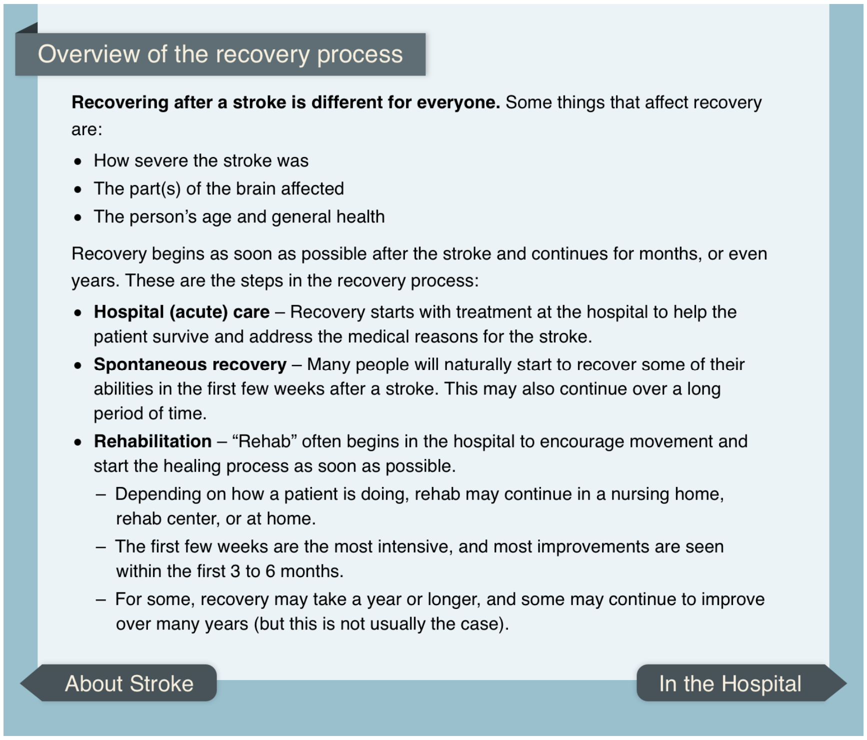

IV. In the Hospital

a. Very early treatments

Early treatments for intracerebral hemorrhage (ICH) or ischemic stroke (IS) are listed on this page (tailored to patient condition). This helps the surrogate decision maker understand what decisions they may be asked to make on the patients' behalf.

Intracerebral Hemorrhage

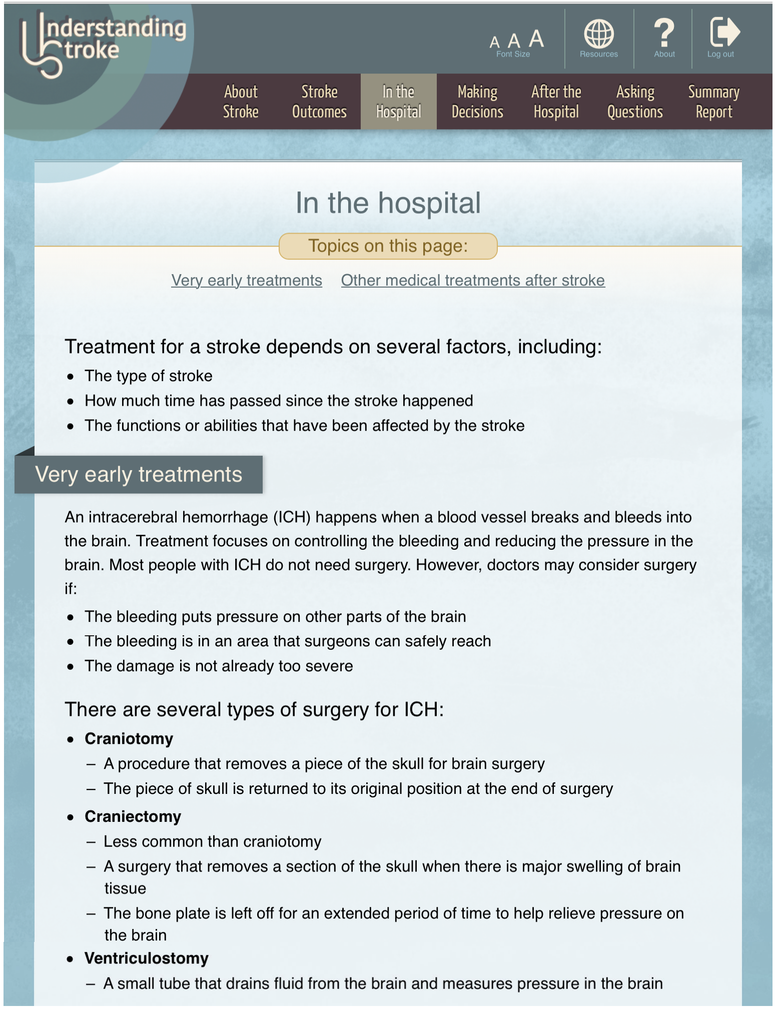

Ischemic Stroke

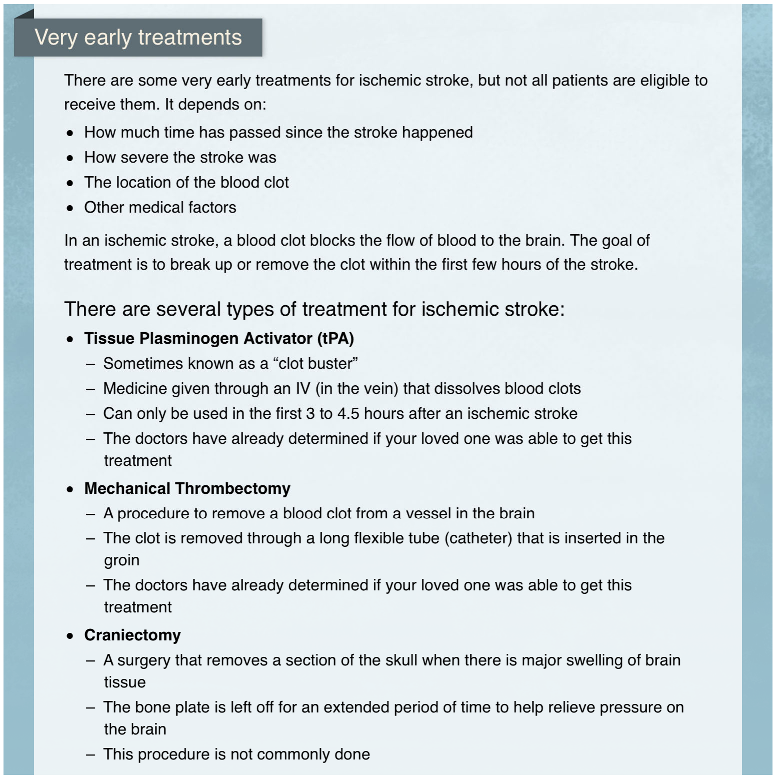

b. Other medical treatments after stroke

Other common medical treatments or key decisions (i.e., DNR orders) for ICH and IS patients are briefly introduced here as well.

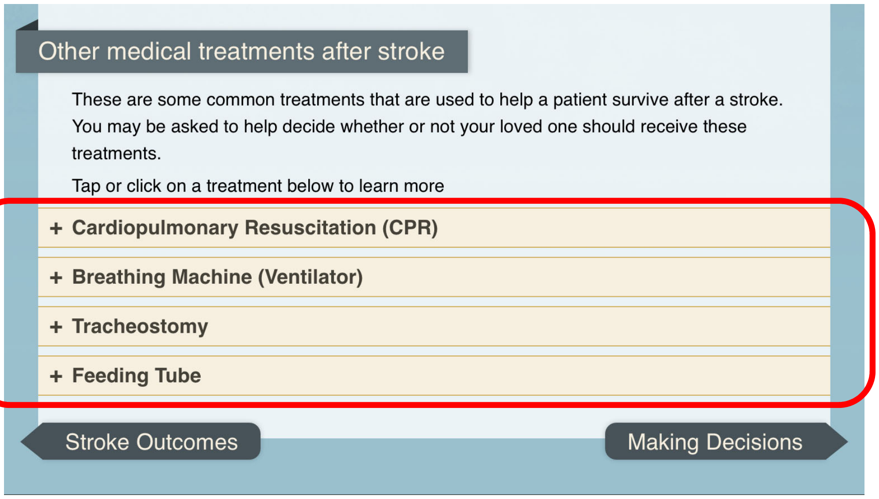

Each treatment is organized into an accordion that can be expanded or closed depending on surrogate interest.

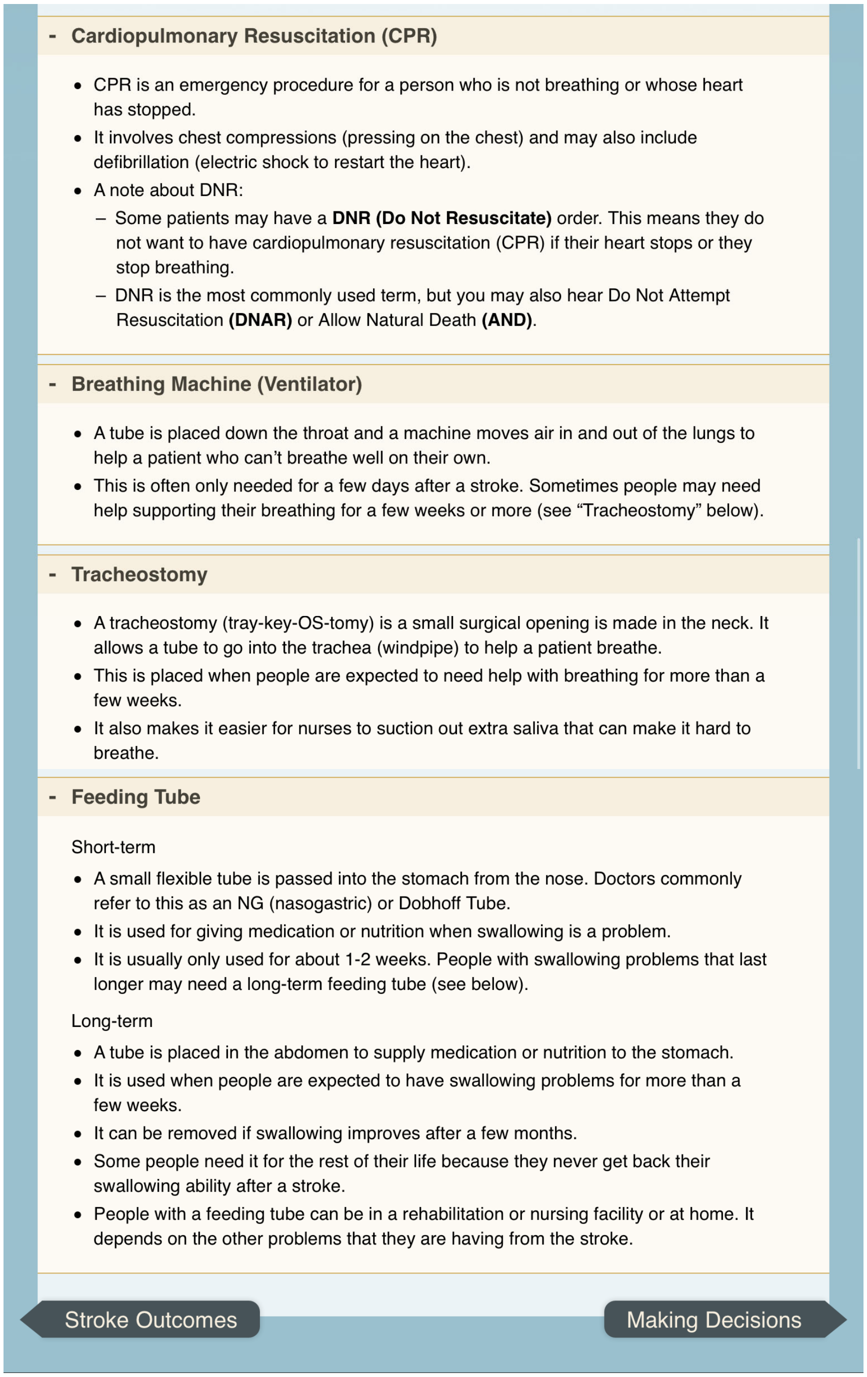

V. Making Decisions

a. Making healthcare decisions for someone else

This section describes what it means to be a surrogate decision maker and how to make healthcare decisions for someone else.

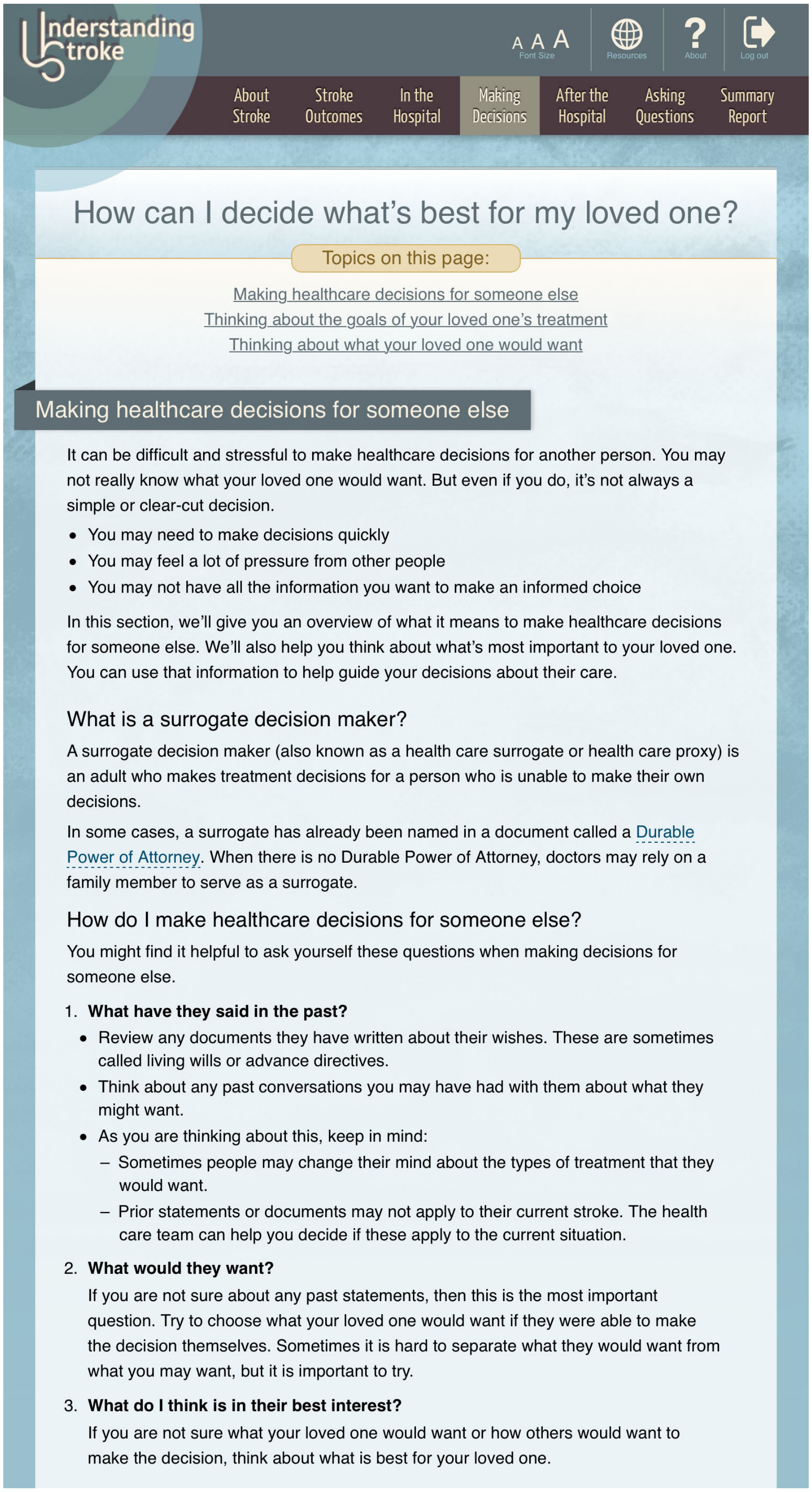

Understanding Stroke

A A A Font Size | Resources | About | Log out

About Stroke | Stroke Outcomes | In the Hospital | Making Decisions | After the Hospital | Asking Questions | Summary Report

How can I decide what's best for my loved one?

Topics on this page:

Making healthcare decisions for someone else
Thinking about the goals of your loved one's treatment
Thinking about what your loved one would want

Making healthcare decisions for someone else

It can be difficult and stressful to make healthcare decisions for another person. You may not really know what your loved one would want. But even if you do, it's not always a simple or clear-cut decision.

- You may need to make decisions quickly
- You may feel a lot of pressure from other people
- You may not have all the information you want to make an informed choice

In this section, we'll give you an overview of what it means to make healthcare decisions for someone else. We'll also help you think about what's most important to your loved one. You can use that information to help guide your decisions about their care.

What is a surrogate decision maker?

A surrogate decision maker (also known as a health care surrogate or health care proxy) is an adult who makes treatment decisions for a person who is unable to make their own decisions.

In some cases, a surrogate has already been named in a document called a Durable Power of Attorney. When there is no Durable Power of Attorney, doctors may rely on a family member to serve as a surrogate.

How do I make healthcare decisions for someone else?

You might find it helpful to ask yourself these questions when making decisions for someone else.

1. **What have they said in the past?**
   - Review any documents they have written about their wishes. These are sometimes called living wills or advance directives.
   - Think about any past conversations you may have had with them about what they might want.
   - As you are thinking about this, keep in mind:
     - Sometimes people may change their mind about the types of treatment that they would want.
     - Prior statements or documents may not apply to their current stroke. The health care team can help you decide if these apply to the current situation.
2. **What would they want?**
   If you are not sure about any past statements, then this is the most important question. Try to choose what your loved one would want if they were able to make the decision themselves. Sometimes it is hard to separate what they would want from what you may want, but it is important to try.
3. **What do I think is in their best interest?**
   If you are not sure what your loved one would want or how others would want to make the decision, think about what is best for your loved one.

b. Thinking about the goals of your loved one's treatment

Given the multitude of life-sustaining treatment decisions that a stroke surrogate decision maker may face (e.g. intubation, resuscitation, feeding tube), Understanding Stroke was designed to frame the decision as setting the overall goals of treatment rather than on any individual treatment or procedure. The overall goals of treatment were adapted from prior work (see manuscript references) and includes Life-Prolonging Treatment, Basic Treatment, and Comfort Measures Only.

Each treatment is organized into an accordion that can be expanded or closed depending on surrogate interest.

Thinking about the goals of your loved one's treatment

Before making specific decisions for your loved one, it helps to think about the overall goal of their medical treatment. While there are different ways to define the overall goals of treatment, it is sometimes helpful to think about this as 3 main categories:

+ Life-Prolonging Treatment

+ Basic Treatment

+ Comfort Measures Only

This table summarizes the 3 categories. You can click or tap on the colored bars above to read more about each category.

	**Life-Prolonging Treatment** Goal: Keep alive as long as possible	**Basic Treatment** Goal: Maintain physical and mental functions	**Comfort Measures Only** Goal: Maximize comfort and relieve pain
Cardiopulmonary resuscitation (CPR) and defibrillation (electric shock to the heart)	X		
Breathing machine (ventilator)	X		
Intensive Care Unit (ICU) care	X		
Intravenous (IV) therapy	X	X	Sometimes used for pain
Hospitalization	X	X	Sometimes
Physical, occupational, or speech therapy	X	X	
Pain relief	X	X	X

- Life-Prolonging Treatment

Goal: **help the person live as long as possible to allow time to recover from the stroke**

There are no limits to treatment and it includes everything a modern hospital has available to keep someone alive, such as:

- Using chest compressions and electric shocks to restart the heart if it stops beating
- Breathing machines (ventilators) if a person cannot breathe well on their own
- Other medical procedures that are done in the intensive care unit (ICU)

- Basic Treatment

Goal: **maintain physical and mental functions and allow time to recover from the stroke**

Some of the more intensive or invasive treatments may be limited. Treatment depends on the person's physical and mental functioning and may include:

- Intravenous (IV) therapies like antibiotics
- Hospitalization
- Physical, occupational, and speech therapy to improve recovery from stroke

Basic treatment **does not include** cardiopulmonary resuscitation (CPR), breathing machines, or intensive care unit (ICU) care.

- Comfort Measures Only

Goal: **maximize comfort and relieve pain**

- Treatment is only used to keep the person comfortable.
- Comfort care **does not include** cardiopulmonary resuscitation (CPR), breathing machines, or intensive care unit (ICU) care.
- Occasionally, intravenous (IV) medications or hospitalization are used.
- There are no attempts made to artificially prolong life.

c. Thinking about what your loved one would want

This section focuses on helping the surrogate clarify the patient's values. A list of situations where a patient might need help with activities of daily living is presented, and the surrogate is asked to pick up to 5 items that may cause their loved one to consider stopping treatment and choosing comfort measures only. A "None of the above" option is also offered for those who feel their loved one would not want to stop treatment under any circumstances. Their choices are recorded and saved into their Summary Report.

Thinking about what your loved one would want

People have different opinions about what makes life worth living. In this section, we'll try to help you clarify what your loved one would want so that you can make better decisions for them.

The list below shows some activities or abilities that can be affected by stroke. These may or may not apply to your loved one.

As you review the list, think about **which situations might affect your loved one's happiness or quality of life so much that they might consider stopping treatment** to extend life and having comfort measures only

- You may find that you think about each item differently, depending on whether the problem would be temporary or longer-term. For now, **try to think about what your loved one would want if they had these problems long term – for a few months or more.**
- The items you select will be included in your summary report. This list is to help guide your conversation with the health care team. It will not be used by itself to make treatment decisions. You can change your mind about these items at any time.

My loved one might consider stopping treatment to extend life (and choose comfort measures only) if, for a few months or more, they…

Select the most important items (up to 5)

- ☐ Were not able to talk, but could still engage in non-verbal communication
- ☐ Had difficulty thinking of words or understanding others
- ☐ Were not able to breathe without the help of a machine
- ☐ Were not able to participate in important hobbies, social, or religious activities
- ☐ Had difficulty thinking clearly or making decisions (e.g. needed help managing finances)
- ☐ Needed help from another person to eat, bathe, or take care of basic bathroom needs
- ☐ Were not able to live on their own or take care of themselves
- ☐ Needed a walker or wheelchair to move around
- ☐ Had to stay in bed constantly
- ☐ Needed a feeding tube to get nutrition
- ☐ Had a lot of discomfort or pain
- ☐ Had to stay in a nursing home or rehabilitation facility
- ☐ Could not move one side of their body
- ☐ **None of the above.** My loved one would want treatment to extend life in all of these situations.

This activity has already been completed and saved to your Summary Report. You can print the Summary Report and bring it with you when you talk to the health care team. If you'd like to save different choices, you can print this page after making your selection. (Go to File>Print in your browser menu.)

In the Hospital

After the Hospital

VI. After the Hospital

a. Rehabilitation settings

A table with rehabilitation options is presented to the surrogate to help them determine what may be the best fit for their loved one. It is also briefly mentioned that there are treatments available to prevent another stroke.

b. Adjusting to life after stroke

Adjusting to life after stroke may be difficult for both the patient and their families. Resources are provided that may help with adjusting to a "new normal".

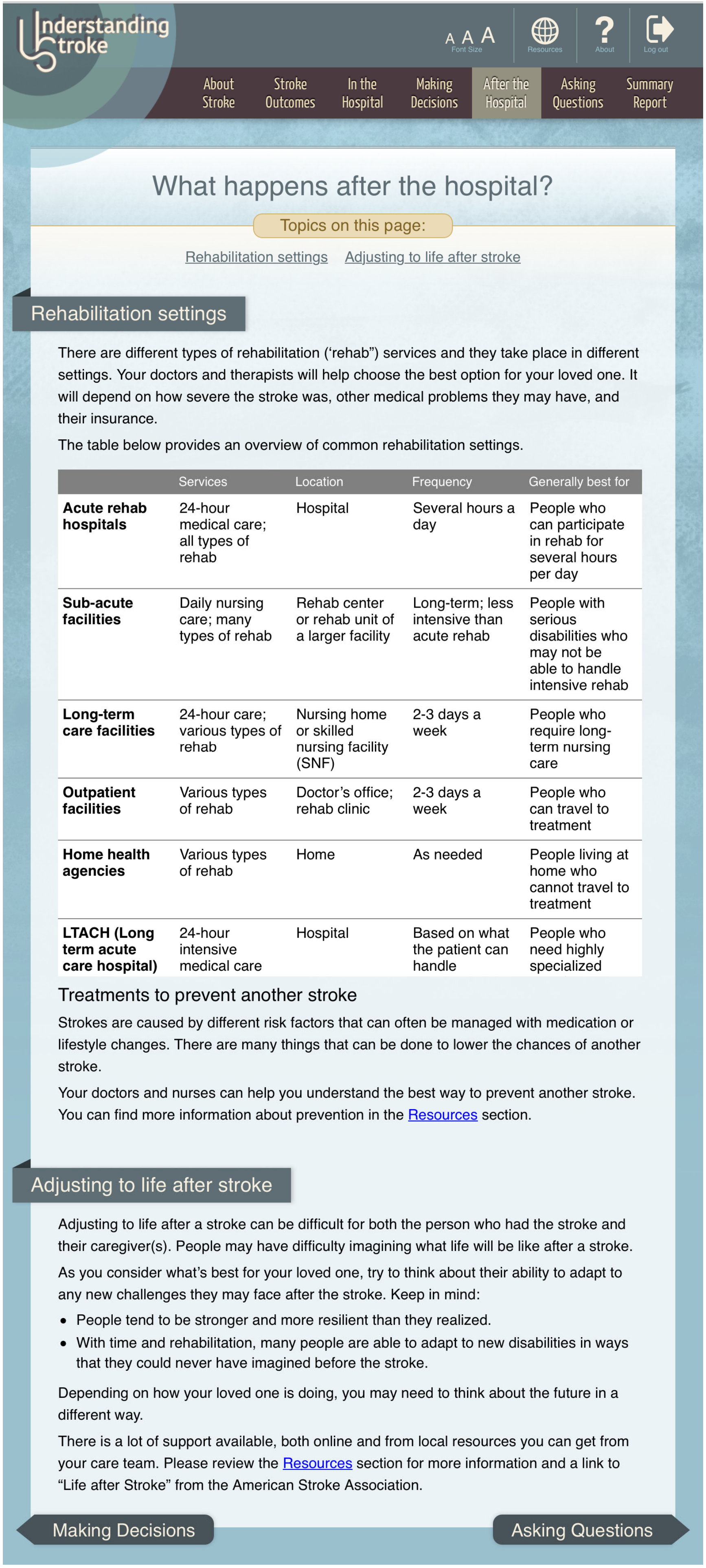

Understanding Stroke

A A A Font Size | Resources | About | Log out

About Stroke | Stroke Outcomes | In the Hospital | Making Decisions | After the Hospital | Asking Questions | Summary Report

What happens after the hospital?

Topics on this page:

Rehabilitation settings   Adjusting to life after stroke

Rehabilitation settings

There are different types of rehabilitation ('rehab") services and they take place in different settings. Your doctors and therapists will help choose the best option for your loved one. It will depend on how severe the stroke was, other medical problems they may have, and their insurance.

The table below provides an overview of common rehabilitation settings.

	Services	Location	Frequency	Generally best for
**Acute rehab hospitals**	24-hour medical care; all types of rehab	Hospital	Several hours a day	People who can participate in rehab for several hours per day
**Sub-acute facilities**	Daily nursing care; many types of rehab	Rehab center or rehab unit of a larger facility	Long-term; less intensive than acute rehab	People with serious disabilities who may not be able to handle intensive rehab
**Long-term care facilities**	24-hour care; various types of rehab	Nursing home or skilled nursing facility (SNF)	2-3 days a week	People who require long-term nursing care
**Outpatient facilities**	Various types of rehab	Doctor's office; rehab clinic	2-3 days a week	People who can travel to treatment
**Home health agencies**	Various types of rehab	Home	As needed	People living at home who cannot travel to treatment
**LTACH (Long term acute care hospital)**	24-hour intensive medical care	Hospital	Based on what the patient can handle	People who need highly specialized

Treatments to prevent another stroke

Strokes are caused by different risk factors that can often be managed with medication or lifestyle changes. There are many things that can be done to lower the chances of another stroke.

Your doctors and nurses can help you understand the best way to prevent another stroke. You can find more information about prevention in the Resources section.

Adjusting to life after stroke

Adjusting to life after a stroke can be difficult for both the person who had the stroke and their caregiver(s). People may have difficulty imagining what life will be like after a stroke.

As you consider what's best for your loved one, try to think about their ability to adapt to any new challenges they may face after the stroke. Keep in mind:

- People tend to be stronger and more resilient than they realized.
- With time and rehabilitation, many people are able to adapt to new disabilities in ways that they could never have imagined before the stroke.

Depending on how your loved one is doing, you may need to think about the future in a different way.

There is a lot of support available, both online and from local resources you can get from your care team. Please review the Resources section for more information and a link to "Life after Stroke" from the American Stroke Association.

Making Decisions   Asking Questions

VII. Asking Questions

This section gives the surrogate some tips on talking to the health care team, as well as a suggested list of questions they may want to ask. A free text field is also offered for them to enter any additional questions. Their choices are recorded and saved in their Summary Report.

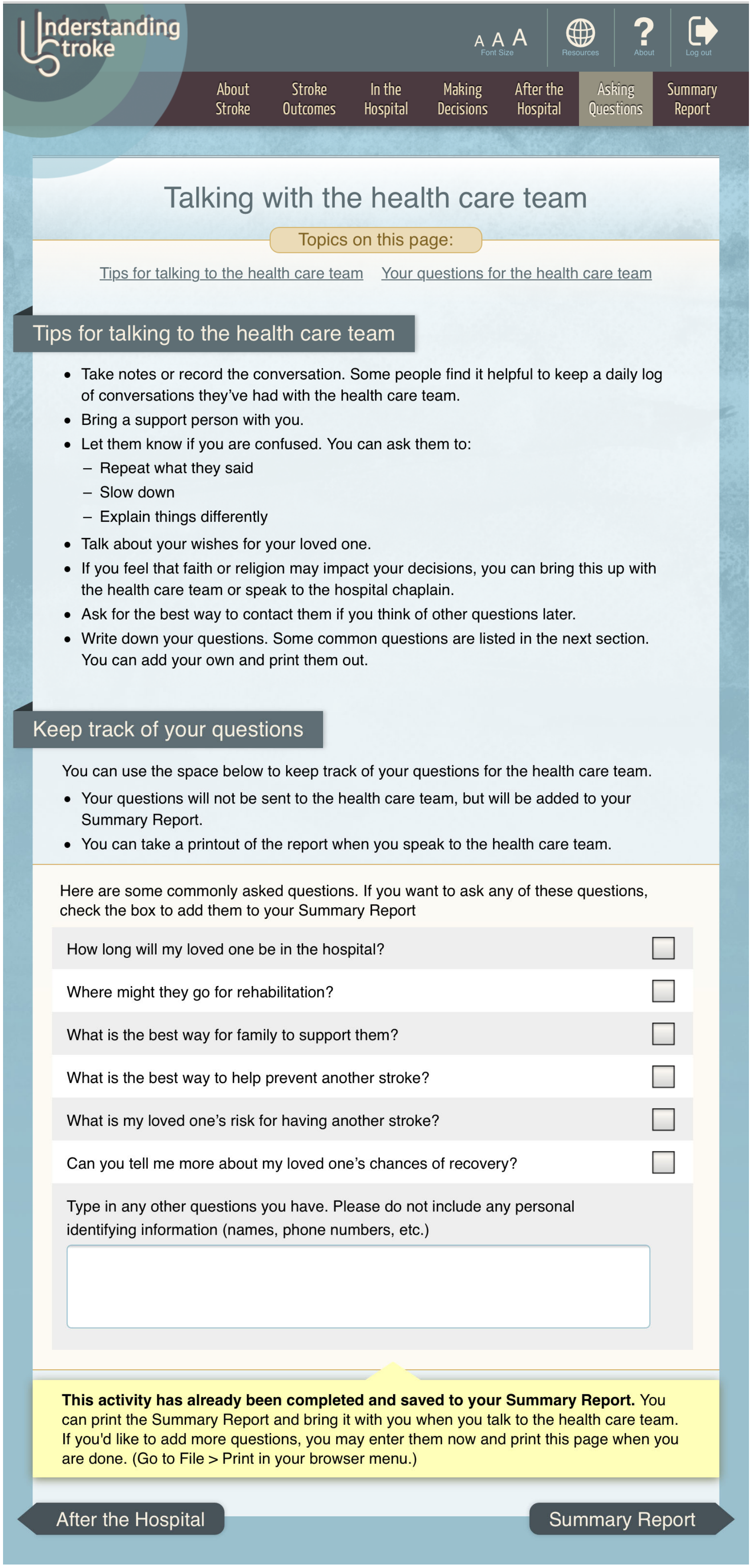

Understanding Stroke

A A A Font Size | Resources | About | Log out

About Stroke | Stroke Outcomes | In the Hospital | Making Decisions | After the Hospital | Asking Questions | Summary Report

Talking with the health care team

Topics on this page:

Tips for talking to the health care team   Your questions for the health care team

Tips for talking to the health care team

- Take notes or record the conversation. Some people find it helpful to keep a daily log of conversations they've had with the health care team.
- Bring a support person with you.
- Let them know if you are confused. You can ask them to:
  - Repeat what they said
  - Slow down
  - Explain things differently
- Talk about your wishes for your loved one.
- If you feel that faith or religion may impact your decisions, you can bring this up with the health care team or speak to the hospital chaplain.
- Ask for the best way to contact them if you think of other questions later.
- Write down your questions. Some common questions are listed in the next section. You can add your own and print them out.

Keep track of your questions

You can use the space below to keep track of your questions for the health care team.

- Your questions will not be sent to the health care team, but will be added to your Summary Report.
- You can take a printout of the report when you speak to the health care team.

Here are some commonly asked questions. If you want to ask any of these questions, check the box to add them to your Summary Report

- [ ] How long will my loved one be in the hospital?
- [ ] Where might they go for rehabilitation?
- [ ] What is the best way for family to support them?
- [ ] What is the best way to help prevent another stroke?
- [ ] What is my loved one's risk for having another stroke?
- [ ] Can you tell me more about my loved one's chances of recovery?

Type in any other questions you have. Please do not include any personal identifying information (names, phone numbers, etc.)

This activity has already been completed and saved to your Summary Report. You can print the Summary Report and bring it with you when you talk to the health care team. If you'd like to add more questions, you may enter them now and print this page when you are done. (Go to File > Print in your browser menu.)

After the Hospital   Summary Report

VIII. Summary Report

The Summary Report collates key information from the website into a report that the surrogate can print out and use in their conversations with the health care team.
The Summary Report includes stroke type, prognosis, results from the values clarification exercise, tips for talking to the health care team, a list of the surrogate's questions, as well as national and regional resources (not pictured).

An example for Ischemic Stroke is shown.

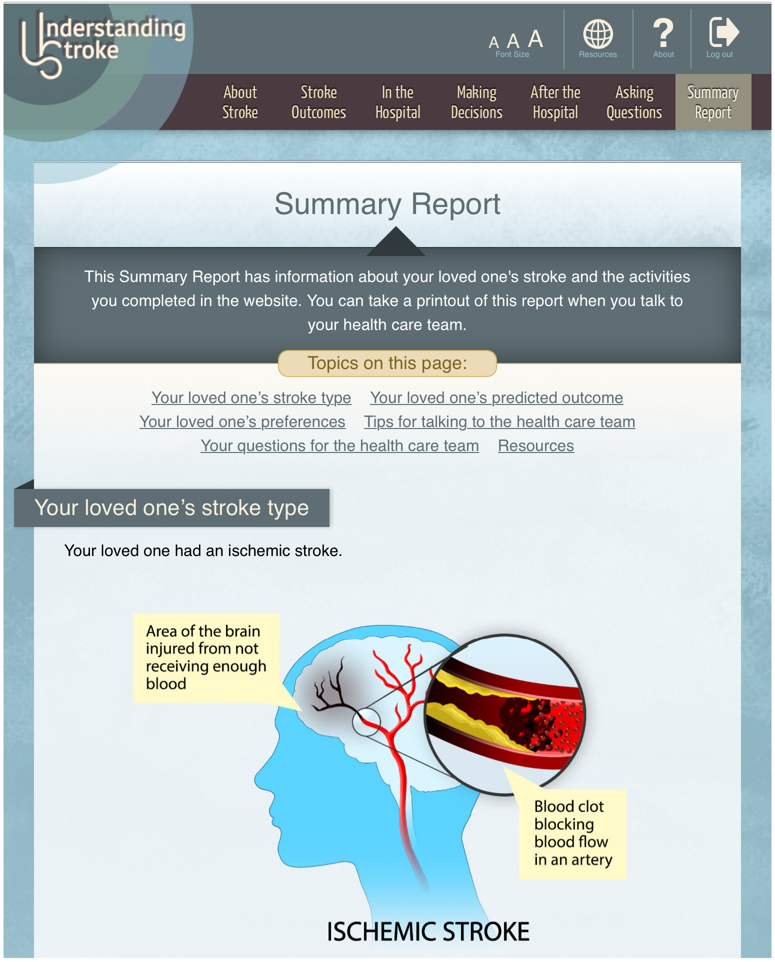

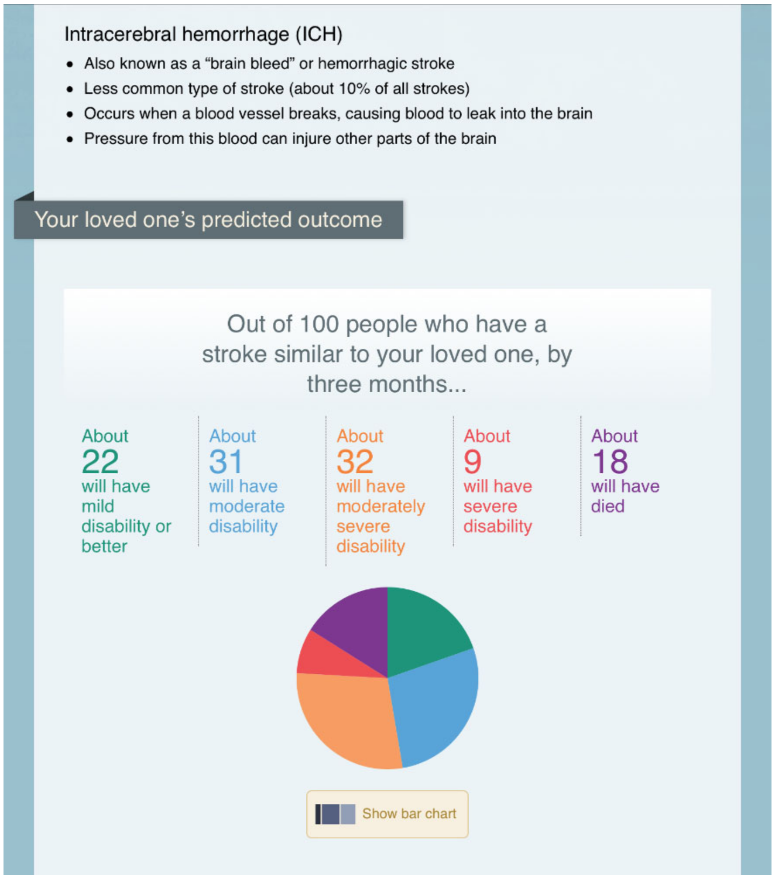

Your loved one’s preferences

These are the situations you felt would affect your loved one’s happiness or quality of life so much that they might consider comfort measures only:

- Was not able to talk, but could still engage in non-verbal communication
- Was not able to breathe without the help of a machine
- Was not able to participate in important hobbies, social, or religious activities
- Needed help from another person to eat, bathe, or take care of basic bathroom needs

Choices above are from the first time this activity was completed

Tips for talking to the health care team

- Take notes or record the conversation. Some people find it helpful to keep a daily log of conversations they’ve had with the health care team.
- Bring a support person with you.
- Let them know if you are confused. You can ask them to:
  - Repeat what they said
  - Slow down
  - Explain things differently
- Talk about your wishes for your loved one.
- If you feel that faith or religion may impact your decisions, you can bring this up with the health care team or speak to the hospital chaplain.
- Ask for the best way to contact them if you think of other questions later.
- Write down your questions. Some common questions are listed in the next section. You can add your own and print them out.

Your questions for the health care team

Other questions or concerns you have:

question ?

Choices above are from the first time this activity was completed
